# The Case of the Wandering Kidney

**DOI:** 10.1155/2013/498507

**Published:** 2013-12-11

**Authors:** David W. Sobel, Brian M. Jumper

**Affiliations:** ^1^School of Medicine, Tufts University, Boston, MA 02111, USA; ^2^Pediatric Urology, Maine Medical Center, Tufts University, Portland, ME 04102, USA

## Abstract

Nephroptosis is a controversial phenomenon well described in the literature. In this case report, we present a patient whose right kidney had “wandered” from its normal anatomic position in the retroperitoneum anteriorly and was in a fixed position anterior to the liver secondary to hydronephrosis. As opposed to the suspected mechanism of nephroptosis, we offer a hydraulic theory as to the origin of the energy required to cause this translocation. The work required to move the patient's kidney was generated by her cardiac output.

## 1. Introduction

Nephroptosis, or “floating kidney,” is a well-known and controversial phenomenon where the kidneys descend bilaterally by a significant distance (traditionally >5 cm or two vertebral bodies on IVU) upon moving from supine to erect [1]. Historically, symptomatic nephroptosis has been treated with a multitude of surgical and nonsurgical modalities, including capsulectomy, fixation with sutures, and abdominal binding with restrictive clothing [2]. More recently, in the twentieth century, nephropexy has been suggested as the definitive therapy for symptomatic nephroptosis. The efficacy of this has been questioned too [3]. In this case report, we describe a patient whose right kidney had “wandered” from its normal anatomic position in the retroperitoneum anteriorly and was in a fixed position anterior to the liver secondary to hydronephrosis. As opposed to the suspected mechanism of nephroptosis, we offer a hydraulic theory as to the origin of the energy required to cause this translocation.

## 2. Case Presentation

The patient was an 87-year-old female who initially presented to her primary care provider in May 2010 complaining of increasing abdominal pain and tightness in the central abdomen for two days with associated nausea, vomiting, and anorexia. Her physician sent her to the emergency department for further evaluation. The patient's surgical history included an open appendectomy in 1960 and her past medical history was significant for hypertension, atrial fibrillation, polycythemia rubra vera, and epilepsy. Her urological history was unremarkable. According to the patient, she had similar episodes in the past few years where she felt occasional bloating and the feeling that something was protruding into her scar, but that pain usually passed within a day.

**Figure 1:**
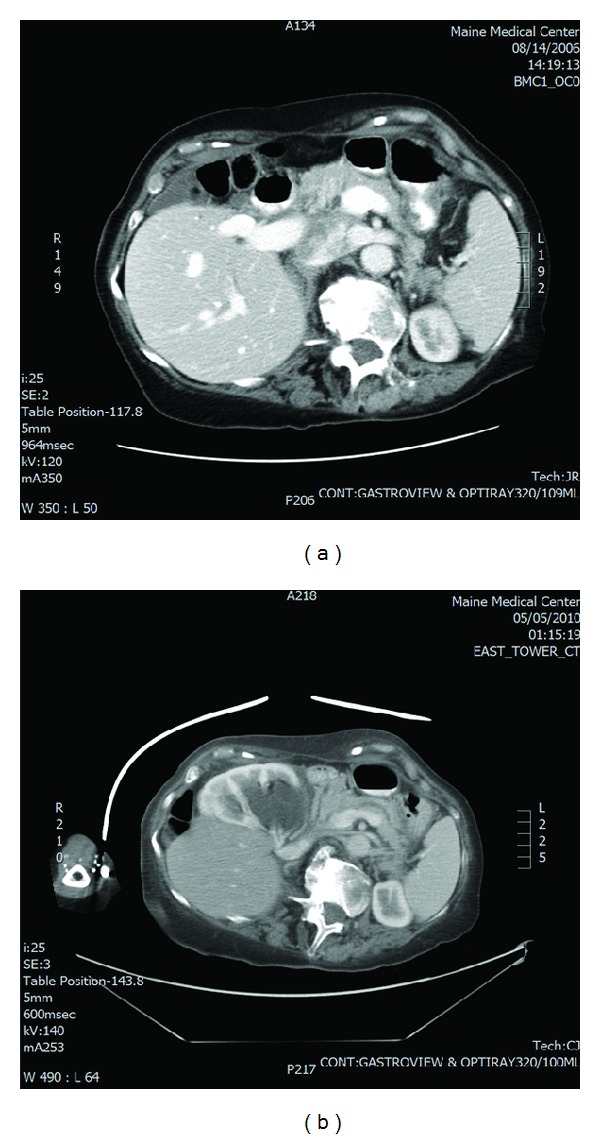
Previous CT scan from 2006 (a) and CT scan from 2010; (b) patient presentation in the emergency department depicting new anterior position of right kidney.

**Figure 2:**
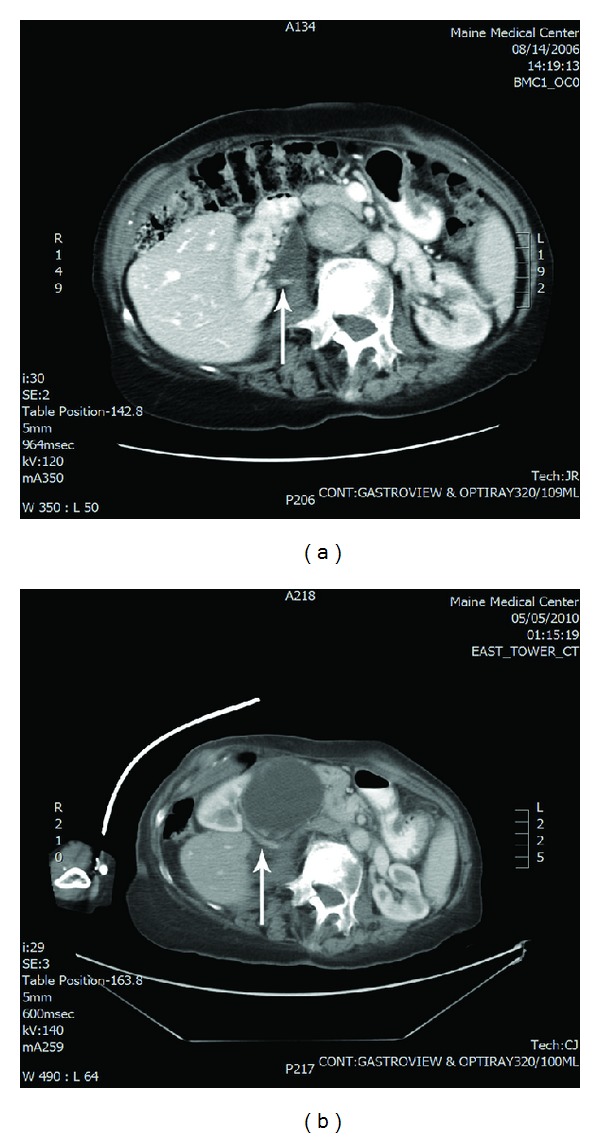
Previous CT scan from 2006 (a) and CT scan from 2010; (b) patient presentation in the emergency department depicting crossing vessel (indicated by arrow).

Upon evaluation in the emergency department, the emergency medicine resident noted a “soft, midline mass, which is tender and is incompletely reducible with moderate increase in discomfort.” A healed 5 cm scar was present in the right abdomen. Bowel sounds were increased, and the mass was initially attributed to a ventral hernia causing the patient's symptoms. An acute abdominal series showed no clear signs of obstruction. A CT scan (Figures 1 and 2) of the abdomen was obtained due to her age and severity of pain and the report from the on-call radiologist noted the following.

“The right kidney is no longer within the renal fossa as seen in 2006, but has changed position and is now anterior to the liver. There is resultant severe hydronephrosis involving the renal pelvis and proximal ureter. This abruptly changes caliber on axial image 38 and the ureter is not well seen inferior to this level. There is symmetric renal perfusion. High attenuation material is seen dependently within the dilated renal pelvis, this could reflect blood products or stone material.” Additionally, there was no diaphragmatic hernia noted.

The urologist on call was then called at approximately two thirty in the morning to assess the patient, and noted in the documentation the following. “A patient with what appears to be UPJ obstruction on the right side that has developed since 2006 when she had a CT scan demonstrating what looks to be a lower pole crossing the arterial vessel. She has since had progressive obstruction that has pushed her renal pelvis posteriorly and her kidney anteriorly up in front of her liver and has displaced her colon. I am concerned that a full surgical repair would require open surgery in this delicate 88 year old and would suggest that she undergo a cystoscopy and right double-J stent placement to assess how she tolerates that.” Approximately 6 weeks following the emergency department visit, a cystoscopy, bilateral retrograde pyelograms, and right double-J stent placement were performed. This ameliorated her symptoms and the stents were removed in a timely fashion.

The patient was seen recently in followup in June 2013 and she stated that she had an additional episode of abdominal pain similar to her presentation to the emergency department and that manual pressure on her right upper quadrant was successful in reducing the mass and relieving the pain. In followup, the right kidney was palpable but nontender. The patient was scheduled for a routine six-month followup to track her progress. Since then, she endorsed self-treating with manual manipulation of the kidney when it has been painful.

**Figure 3:**
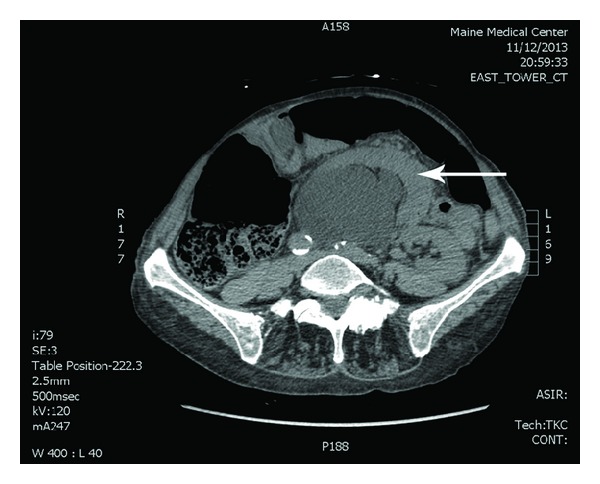
CT scan from November 2013 performed in the emergency department depicting right kidney (indicated by arrow) anterior to left kidney with extreme hydronephrosis.

**Figure 4:**
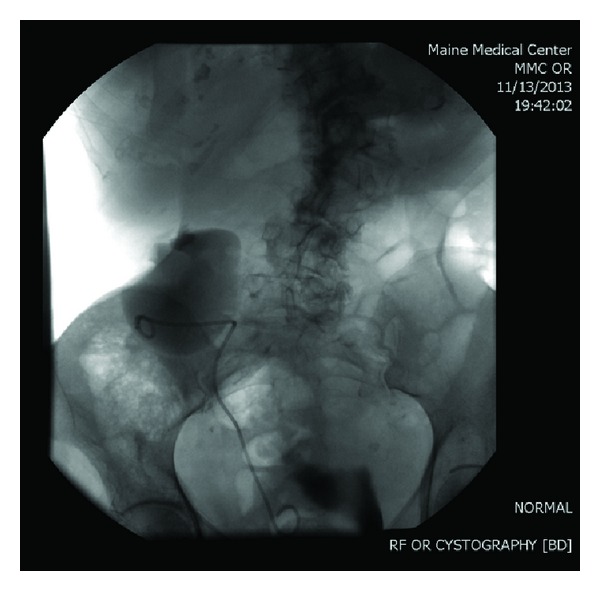
Right retrograde pyelogram conducted the following day indicating translocated right kidney now in the right lower quadrant with double-J stent in place.

In November 2013, however, the patient again presented to the emergency department with the same abdominal pain. A noncontrast CT scan was performed and the patient's right kidney had now translocated across the midline to a position anterior to her left kidney with extreme hydronephrosis present (Figure 3). The following day, a retrograde pyelogram (Figure 4) was performed showing that the right kidney had moved back to the right lower quadrant. A double-J stent was placed to reduce the hydronephrosis and subsequently her symptoms resolved. She is expected to follow up for repeated imaging and stent removal.

## 3. Discussion

During the sixth through the ninth week of embryologic development, the kidneys ascend to a lumbar site, just below the adrenal glands. The exact mechanisms responsible for renal ascent are not known, but a combination of embryonic differential growth, vascular supply changes, and regression of transient embryonic structures may all contribute to this phenomenon [4]. This ascent does not imply that the kidney defies gravity to attain its ultimate position in the body.

Nephroptosis is a common finding caused by muscular contraction of the diaphragm during respirations or by assuming an upright posture, allowing gravitational force to lower the kidney from its usual position. Over the time period from 2006 to 2010, our patient experienced progressive scoliosis and a shortening of her stature. A previous nonobstructing lower pole vessel to her right kidney then caused the compression of her proximal ureter, leading to a hydronephrotic change in her collecting system. This hydraulic lift pushed her kidney to its ultimate position in the right upper quadrant, anterior to her liver as seen on her sequential CT scans. To our knowledge, this “wandering kidney” phenomenon has not been previously described in the literature.

In our patient's condition, the result of her right kidney's journey anterior to her liver was caused by a series of events, which were powered by energy from her heart in the form of cardiac output. For urine production to occur, the glomerular filtration is a product of the series of forces favoring and opposing the process. The main driving force is the hydrostatic pressure in the glomerular capillaries, and the filtration is opposed by the hydrostatic pressure in the glomerular capsule and the osmotic pressure attributable to the plasma proteins [5]. Thus, without glomerular filtration, urine production, and eventual hydronephrosis, this patient's kidney could not have traveled to this unique location. The work required to accomplish this movement was generated by her cardiac muscles.
